# Characterizing physical activity and food urban environments: a GIS-based multicomponent proposal

**DOI:** 10.1186/s12942-016-0065-5

**Published:** 2016-10-04

**Authors:** Alba Cebrecos, Julia Díez, Pedro Gullón, Usama Bilal, Manuel Franco, Francisco Escobar

**Affiliations:** 1 Social and Cardiovascular Epidemiology Research Group, School of Medicine, University of Alcalá, Alcalá de Henares, Madrid, Spain; 2 Department of Geology, Geography and Environmental Sciences, University of Alcalá, Calle Colegios 2, 28801 Alcalá de Henares, Madrid, Spain; 3 Escuela Nacional de Sanidad, Instituto de Salud Carlos III, Madrid, Spain; 4 Department of Epidemiology, Johns Hopkins Bloomberg School of Public Health, Baltimore, MD USA

**Keywords:** Synthetic index, Geographic Information Systems, Healthy food availability, Physical activity, Obesogenic environments

## Abstract

**Background:**

Healthier urban environments influence the distribution of cardiovascular risk factors. Our aim was to design and implement a multicomponent method based on Geographic Information Systems to characterize and evaluate environmental correlates of obesity: the food and the physical activity urban environments.

**Methods:**

Study location comprised a socio-demographically average urban area of 12 contiguous census sections (≈16,000 residents), in Madrid, Spain. We conducted on-field audits on all food stores and street segments. We designed a synthetic index integrating continuous measures of both environments, by kernel density analyses. Index ranges from 0 to 100 (least-most healthy).

**Results:**

We found a heterogeneous distribution with 75 and 50 % of the area scoring less than 36.8 and 25.5, respectively. Census sections of study area were categorized by Jenks intervals as high, medium–high, medium–low and low. 41.0 % of residents lived in an area with a low score, 23.6 % medium–low and 31.1 % medium–high and 4.2 % in a high.

**Conclusion:**

The proposed synthetic index may be a relevant tool to inform urban health interventions, providing a feasible way to integrate different measures of barriers and facilitators of healthy urban environments in terms of food and physical activity.

## Background

The obesity epidemic is one of the main public health concerns of the present century [1]. Prevalence of overweight and obesity in European countries ranges from 45 to 67 %. Spain presents some of the highest levels of overweight (60.9 %) and obesity (23.7 %) in Europe [1].

The limited success of current individual-level based strategies shows the need for new approaches based on population-level determinants of obesity [2]. These approaches focus on affecting the fundamental causes [3] of the distribution of risk factors in the whole population [4]. These fundamental causes were called *mass influences* by Rose [4] and are mostly environmental or social factors at several levels. There is a large and renewed interest in these fundamental causes, especially at urban contexts, and particularly at neighborhood level [4–8].

Much of this renewed interest on neighborhood research in chronic diseases focuses on cardiovascular diseases, diabetes mellitus, and obesity [8–10], given that poor access to healthy foods and limited opportunities for physical activity are related to potentially health-relevant neighborhood physical and social environments. As the place of residence is associated with socioeconomic status, neighborhood characteristics can contribute significantly to health inequalities [9, 10]. Unlike other studies focusing on access inequalities to healthy areas as green spaces [11], healthy food environment [8] or health facilities [10], our focus is based on neighborhood characterization as the next step within a wider research.

Body weight regulation depends on multiple factors, such as physical activity and healthy eating [12]. The contextual determinants of physical activity are complex and multifaceted, but can be roughly classified into transport-related physical activity and leisure-time physical activity influences. The determinants of active transportation relate to walking and biking and include features such as quality of pavements, safety, mix land use, destinations or connectivity [13]. Contextual influences of leisure-time or recreational physical activity include sports facilities and green spaces [14]. For this work we took into account the contextual determinants related to walkability. Contextual determinants of healthy eating include all aspects of the local food environment that influence dietary behaviors [15]. Food stores and their associated accessibility and availability of healthy foods have been previously shown to affect dietary behaviors [16].

The literature on the associations between contextual determinants of physical activity and healthy eating is mixed. The diversity of methodologies used and the results obtained [17–19] highlight the complexity of the chain of causation linking contextual factors and different chronic diseases, as well as the challenges inherent on measuring complex social phenomena [20]. Among these challenges there is the intertwining of environmental features: physical activity environments and food environments are not isolated but rather the result of social forces that affect neighborhoods [21].

Much of the previous research has focused solely on one factor in isolation, such as walkability [22] or healthy food availability [23]. Moreover, the strong correlation between physical activity and dietary behaviors calls for strategies that tackle sedentary and unhealthy choices concurrently [24–26]. Interventions may be ineffective if only focused on promoting physical activity, ignoring a food environment which may promote unhealthy foods [24]. Thereby, there is a need of an integrated approach to understand contextual factors of both environments.

A potential promising avenue to operationalize the contextual determinants of obesity is to aggregate measures of both physical activity and diet determinants. Previous studies have aggregated urban context indicators in a synthetic index, finding significant correlations with health outcomes [18, 27, 28]. Kelly-Schwartz et al. [28], found a significant association between a composite index (county sprawl index) and obesity, but not between their components and health outcomes [28].

Geographic Information Systems (GIS) are rapidly becoming a relevant part of the panoply of methods adopted in Public Health research [29]. GIS is a well-suited tool to define healthy urban environments allowing to integrate data from different sources and scales, both spatial and non-spatial. Our objective is to design a multivariable tool based on GIS to integrate information from the physical activity and food environments to better characterize obesogenic environments in urban areas.

## Methods

This study was conducted within the multidisciplinary Heart Healthy Hoods project [30]. The main objective of this European project is to analyse the impact of the physical and social urban environment in relation to residents’ cardiovascular health in Madrid, Spain.

### Study area

Madrid is the capital city of Spain, located in the central area of the country with a population of 3,186,595 habitants [31]. Madrid Metropolitan Region has around 6.5 million residents, the third-largest in Europe, after London and Paris. The City of Madrid is administratively divided into 21 districts which, in turn, are divided into 2412 census sections, the smallest administrative area for the Spanish Census (population~ = 1000–1500 per census section).

In order to conduct our study in an area that was not extreme in sociodemographic or urban form terms, we selected these 12 census sections using the Median Neighborhood Index (MNI) [32]. This method selects clusters of census sections that are on average closest to the median neighborhood in four variables: % above 65 years of age or older, % with low education, % foreign-born and population density. More details on this method can be found in Bilal et al. [32] supplementary material.

**Fig. 1:**
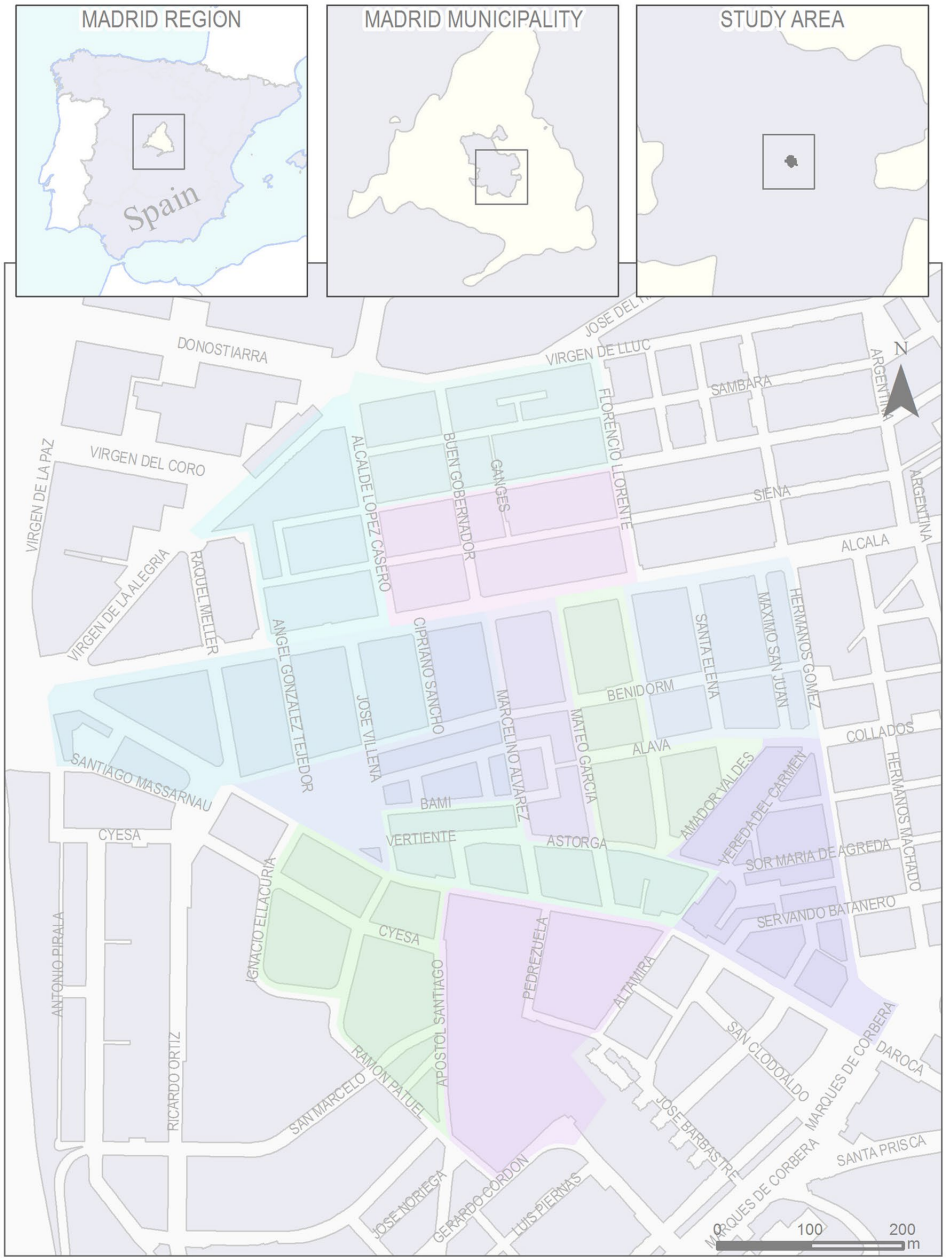
Map of study area. *Colored areas* represent census sections within the pilot study area

Study area is located in the southern part of the district of Ciudad Lineal, adjacent to the city ring road (M-30) (see Fig. 1). This area has an extension of approximately 42 hectares with a total population of 14,980 residents [31]. The study area was developed in the early twentieth century. At that time it was part of the municipality of Canillas and was not incorporated to the City of Madrid until 1955, when the main city incorporated all its surrounding municipalities [33]. The area has received a considerable influx of migration from other rural areas from Spain, especially coincident with the rural exodus of the 60s. Both urban morphology and building structure is relatively homogeneous throughout the area. Most buildings are residential and rank from three to nine stories being the vast majority five stories tall. Given the small size of each census section (~1000–1500 people), there is some random variability in socio-demographic as well as population density within the area, mostly related to the height of the buildings (and hence differential residential density).

According the 2014 Health Report of Madrid City Council [34], 41.2 % of the population presents overweight or obesity, 30.1 and 11.2 % respectively. Although these values are below national measures, 38.4 of overweight 18.2 % of obesity conforming the last National Health Survey, they values remain alarmingly high. Regarding risk factors, the *Risk Factors Surveillance System Associated with Noncommunicable Diseases in Adult Population* of 2013 [35], indicated that 49.2 % of Madrid citizens affirm to be sitting most of their working time and that 73 % were inactive at leisure time. Regarding diet, Madrid citizens eat in average 1.2 rations of fresh fruit and 1.1 rations of vegetables per day. These rates are far from the recommendations of 5 rations per day [36].

### Characterizing the urban environment

#### Food environment

We measured healthy food availability by conducting in-store audits within all food stores present in the study area. We found 40 retail food stores within the selected census sections, which were classified by store type as: corner stores (small stores with a low variety of items and generally no fresh products); grocery stores (midsized stores with higher variety and presence of fresh products); supermarkets (large stores with highest variety and presence of fresh products); specialty stores (greengrocers, butcheries, fishmongers and bakeries); gas stations; and convenience stores (long opening hours and no presence of fresh products). More details of these measurements properties have been published in Bilal et al. [32].

Trained data collectors used an abbreviated version of the Nutrition Environment Measures Survey in Stores (NEMS-S), developed and validated by Glanz et al. [23]. The NEMS-S has been used in several contexts, including the US [37] and Brazil [38]. The abbreviated version was developed by the Johns Hopkins Center for a Livable Future for an assessment of Baltimore’s Food Environment [39]. This instrument examines the availability of healthy options versus less-healthier options over 12 types of foods, such as skim/low-fat milk (vs whole milk), 100 % fruit juice (vs juice drinks), lean ground beef (vs regular), skinless chicken (vs regular), whole grain bread (vs refined bread), or low-regular cereals, as examples [23]. From these surveys, we produced a Healthy Food Availability Index (HFAI) for each food store. Therefore, we looked at 12 food groups: milk, juice, fruits, vegetables, meats, chicken, seafood, canned goods, frozen foods, packaged foods, bread and cereals. The HFAI score in this study could range from 0 to 27.5 points, with a higher score indicating a greater availability of healthy foods [37].

#### Physical activity environment

The Systematic Pedestrian and Cycling Environment Scan (SPACES) [40] is an observational audit of features of the built environment that can influence walking and cycling along a street network. We adapted this audit tool to the Madrid (M-SPACES) environment and conducted a validity and reliability study before [41]. For the purposes of this study, and due to the residual use of bikes in the area, only walkability measures were considered.

A trained researcher audited all street segments of the study area (n = 145 segments) by foot. A street segment is defined as one section of a street that runs between two intersections. It is often used as the basic observation unit in neighborhood or community analysis. Items of the M-SPACES tool are then added up to four domains: functionality, safety, aesthetics and destinations. These, in turn, can be added to compute a walkability score for each street segment (ranging from 0, least walkable, to 1, most walkable). Main audited characteristics were functionality, safety, aesthetics and destinations. More details on this audit tool and its measurements properties have been published before in Gullón et al. [41], and Bilal et al. [32].

#### Spatial datasets

Contextual information on the study area was collected from the Spanish National Mapping Agency and Spanish National Spatial Data Infrastructure, allowing us to generate a georeferenced database to integrate and map the results from the food and physical activity environment assessment. Administrative boundaries (district and census sections) and street networks were collected in vector polygon and line formats, respectively. We also used orthophotograpy of the study area obtained from the Orthophotography Air National Plan.

ArcGIS 10.1 software was used to integrate, standardize and manage these datasets. First, all information was projected to a common system (ETRS89 UTM 30N). The physical activity environment data (collected with the M-SPACES tool) was associated with the street network layer by a relational join. The food environment data (collected with the abbreviated NEMS-S tool) was integrated in the system using a point-based layer with a relational join. All other layers (administrative boundaries, blocks and orthophotos) were introduced to the final maps as reference information.

### Geospatial analysis

**Fig. 2:**
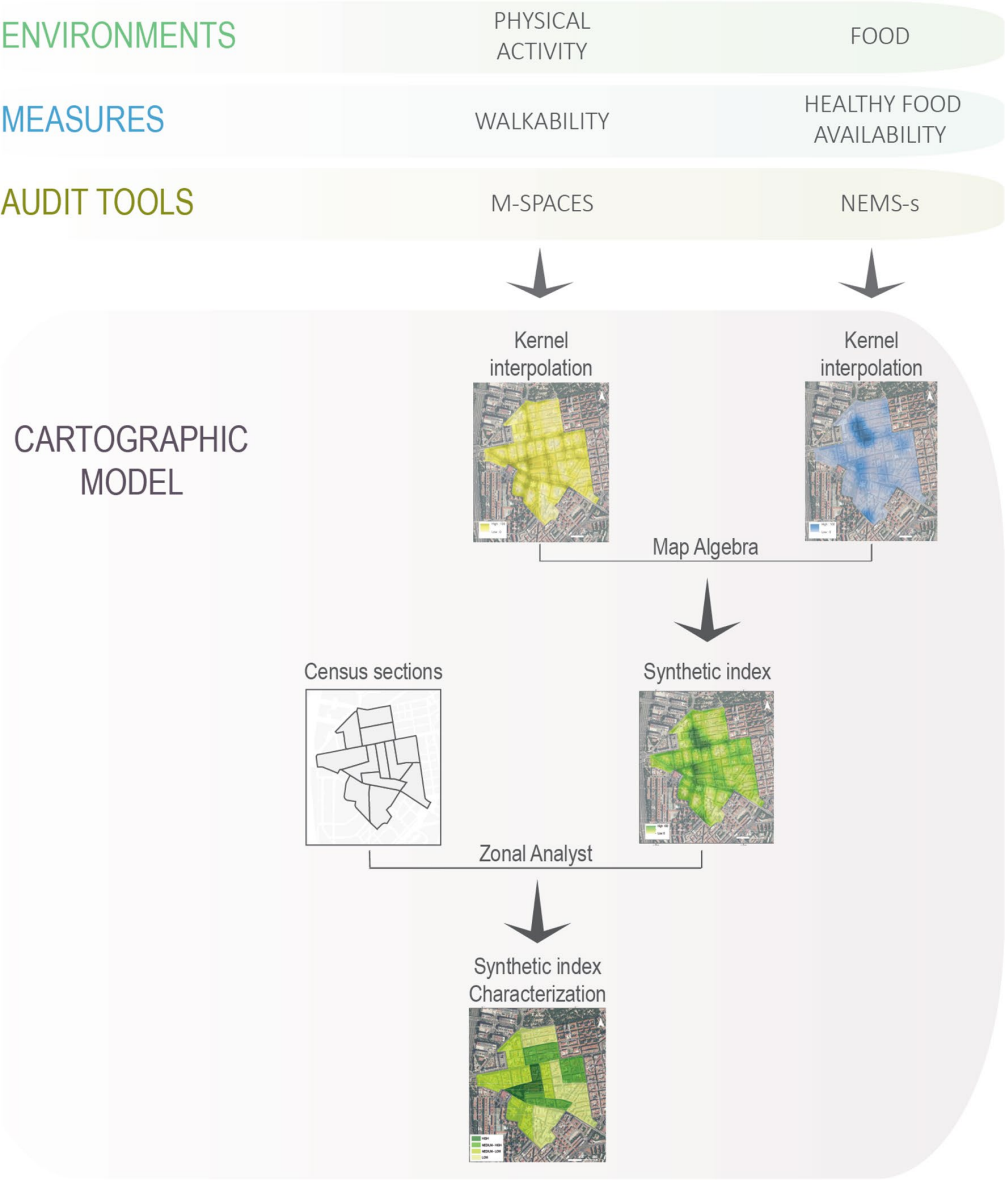
GIS model for the construction of a spatial integrated index on walkability and food environment. Kernel interpolation of the Healthy Food Availability index (HFAI) was created with data from direct observation of food stores measured with the Nutrition Environment Measurement Surveys in Stores (NEMS-S) audit tool. Kernel interpolation of walkability was created with data from direct observation of each street segment measured with the Madrid Pedestrian and Cycling Environment Scan (M-SPACES) audit tool

The aim of this study was to integrate data on the physical activity and food environment in characterizing the urban environment by using GIS. Figure 2 summarizes our approach. In summary, we converted line and point data, linked to physical activity and food respectively, into surface-based data on the whole study area, as a means of facilitating integration of both environments into a single surface. Only after the measures of both environments were known at pixel level (the minimum spatial unit of the newly created surface), a map algebra-based arithmetic operation to combine both measurements was possible. Finally, a categorization of the combining results was applied in order to ease interpretation.

These steps are detailed as follows; First, line data (walkability index for each street segment) and point data (healthy food availability index for each food store) was extended to the whole study area by applying kernel density estimation (KDE), resulting on a pixel-based surface. Both walkability and health food availability indices could have been kept under their original geometric form, line and point respectively. However, a surface-based approach was adopted in order to facilitate future data integration with additional data. Being most statistics aggregated under administrative boundaries, the integration of line or point-based data presents more inconveniences than the assumed ones produced by the extension of the information to the whole surface. In addition, an eventual proposal of a combined index including information on green areas reinforced the surface-based solution.

KDE fits a mathematical surface (composed of pixels) with a normal distribution over each point based on (a) the value empirically collected for each point, and (b) the distance from each location in the surface to all points in the area within defined radius or bandwidth. Essentially, the value of each point is smoothed over the study area producing a density value that will be the highest at the location of every point, and decaying from there with distance using a defined bandwidth [42]. KDE is widely adopted in spatial analysis where input data present different geometric forms and it is of interest the integration of such data with other variables collected on the same territory.

We use de KDE integrated in ArcGis 10.1 software which employs the quadratic Kernel function of Silverman [43]:

$${\hat{\text{f}}}\left( {\text{x}} \right) = \frac{1}{\text{nh}} + \mathop \sum \limits_{{{\text{i}} = 1}}^{\text{n}} {\text{K}}\left( {\frac{{{\text{x}} - {\text{x}}_{\text{i}} }}{\text{h}}} \right)$$

where K is the quadratic Kernel function defined by $K\left( x \right) = \frac{3}{4} \left( {1 - x^{2} } \right), \quad x \le 1$, “x” is the point at which density is estimated, “x_i_” is the value of the variable in the case “i”, “n” is the number of cases and “h” is the bandwidth. The basic idea consists calculated for specific points, the averaged sum (hence the estimator involves summing over “n” and then divide by this value) of Kernels centred on the observations.

This spatial analysis allows weighting each component by their associated attributes, in our case the HFAI and M-SPACES scores. For example, if the component has associated value attribute equal to 3, the case counts as 3 cases. Thus, density value in each pixel of the output image is calculated summing the values of all overlapping kernel surfaces. All surfaces were generated with a pixel size of 3 m. We used a bandwidth of 100 m, given that the average distance from one food store to the closest food store was around half that length (improving smoothing). A static bandwidth was used because of the small study area and the homogeneous population density distribution [42].

The cartographic model presented in Fig. 2 shows the development of both continuous density surfaces: one from the food stores layer weighted by the value of HFAI; and the other from the street segments layer weighted by the scores obtained from the M-SPACES audit.

After generating both surfaces, we performed a map algebra analysis. First, we homogenized data in a range from 0 to 100, to make them comparable with each other. The operation adopted for map algebra was a local unweighted average that computes an average of pixels at the same location in both the physical activity and food environment surfaces, treating both environments with equal weight, generating the synthetic index. To fully integrate the synthetic index into the geographic context of the area, we assigned each census section an obesogenic (synthetic index) value. For this, we used zonal analysis that calculates a single output value for each census section averaging all pixels that fall within each area. To improve the interpretability of our results, we categorized census sections into four classes according to their value in the synthetic index (high, medium–high, medium–low and low). For this, we used the Jenks intervals (or natural breaks) approach that reduce the variance within classes, while maximizing the variance between them.

## Results

**Fig. 3:**
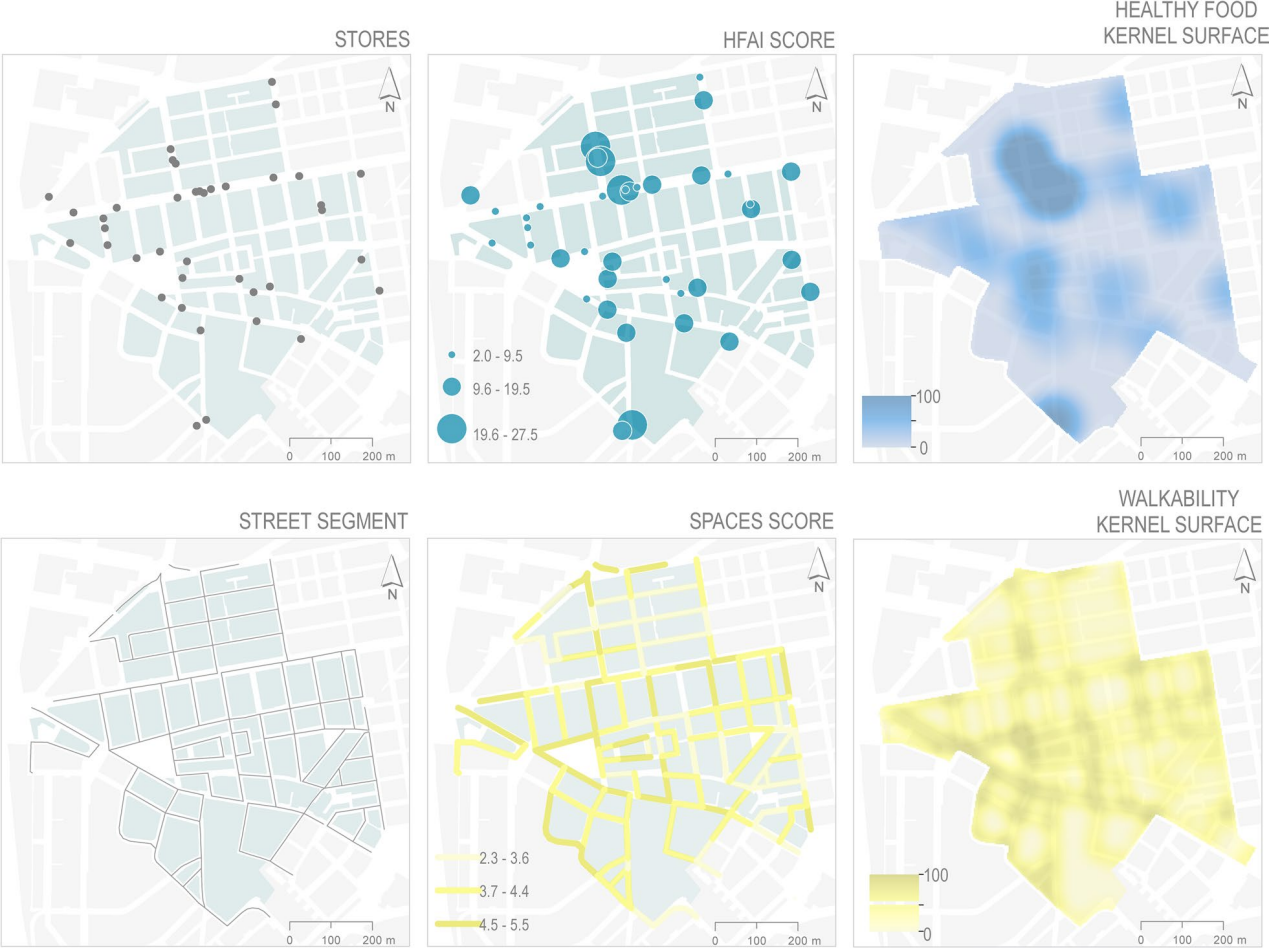
Development of continuous KDE variables from direct observation data. On the *left*, is depicts the calculating for the food environment starting with the location of all stores, continues with the Healthy Food Availability Index (HFAI) score for each one, and the next is the KDE surface weighted by HFAI score. On the *right*, the development for the physical activity environment. Above the location of all the street segments, continues with Pedestrian and Cycling Environment Scan (SPACES) score by each one. And in front the KDE weighted by the SPACES

Figure 3 shows the calculated KDE surface obtained for the food and physical activity environment. Regarding the food environment, the figure shows a concentration of food sales scored with high HFAI values in the North and South ends of the study area, with patches of medium–high density of HFAI distributed throughout the area. Most stores with high HFAI were quite close to each other and mostly located along important roads, creating “islands” of healthy foods. Stores with low HFAI were distributed more evenly creating “healthy food deserts”. Regarding the physical activity environment, the surface resulted from the M-SPACES showed highest values at streets intersections, on streets with wide sidewalks, and in the surroundings of squares and parks. In consequence, the greater the number of intersections, the greater the walkability of the area.

**Fig. 4:**
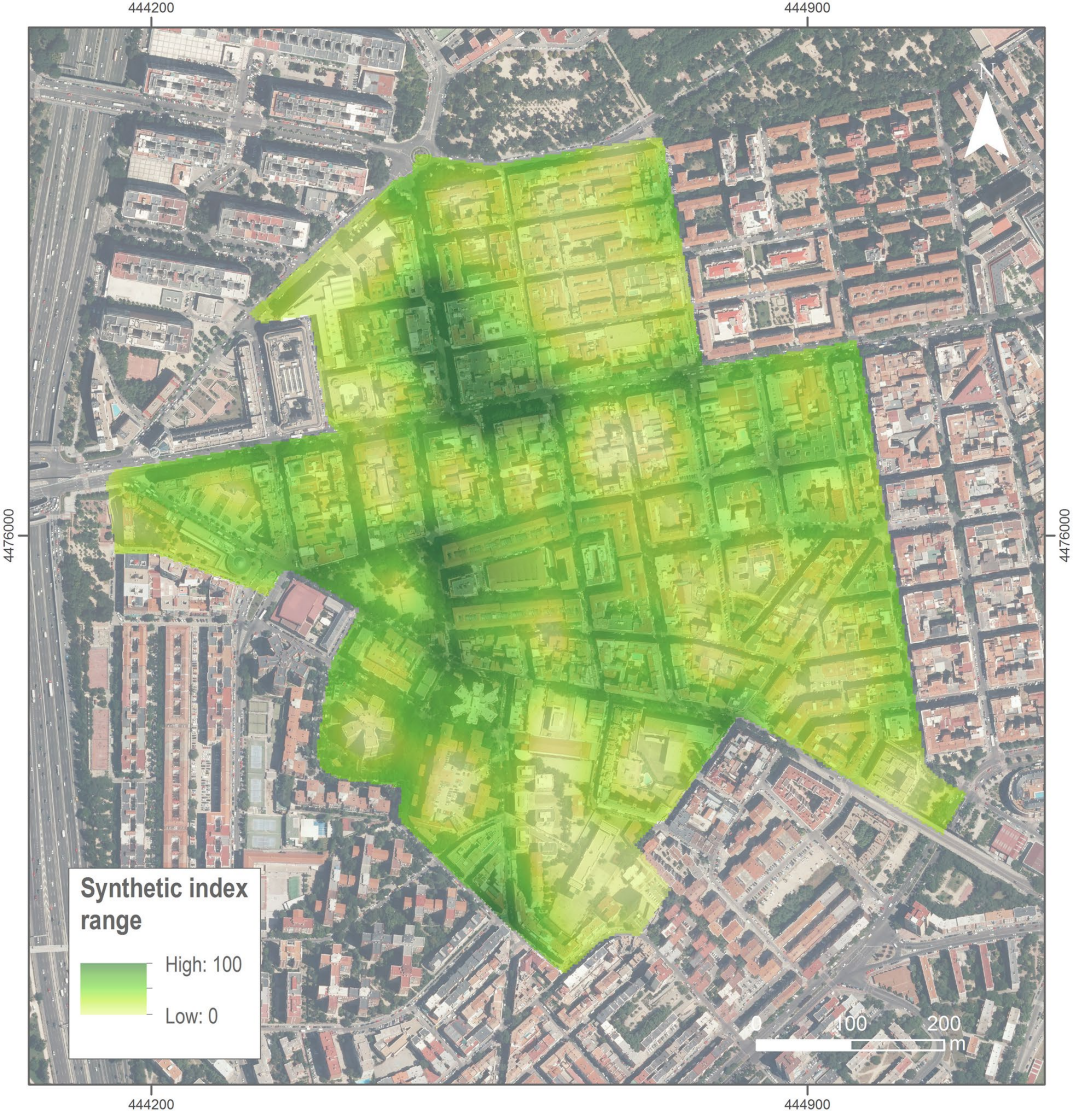
Continuous synthetic index surface. This surface is the local average of the pixels of walkability KDE and the pixels of food availability KDE. The size of pixel is 3 × 3 m and the bandwidth selected to the smoothing was 100 meters

**Fig. 5:**
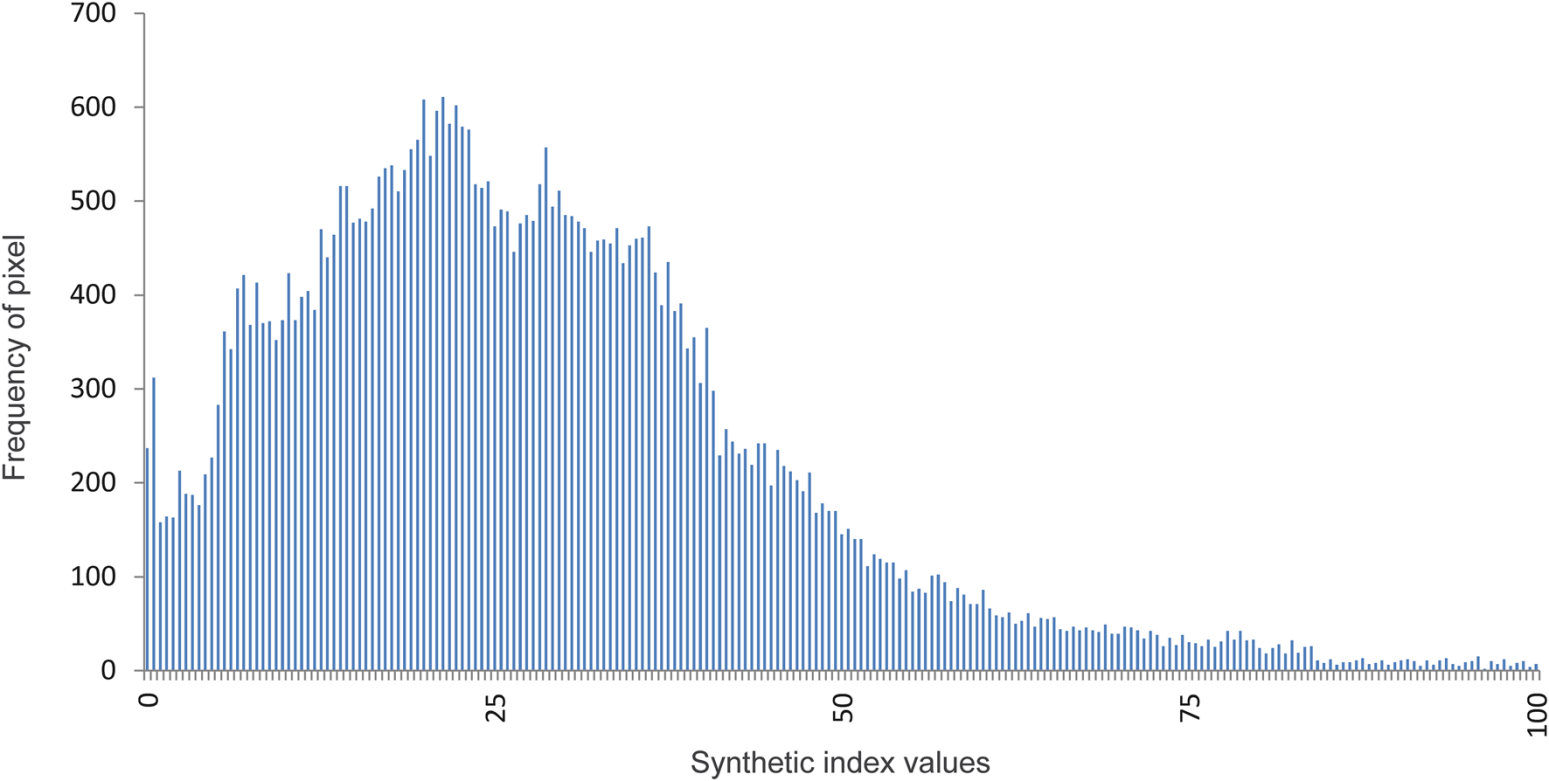
Histogram of synthetic index surface. It depicts the frequency of pixel values of the study area with a range from 0 to 100 with higher scores indicating a healthier environment

The synthetic index surface resulted from averaging the food and physical activity environment is depicted in Fig. 4. Figure 5 shows the distribution of values of the synthetic index. This distribution is right skewed, with a median score of 25.4 (IQR 15.4–36.9) and a mean score of 27.7. Around 75 % of the area is below 36.8 of the index score and half of the area below 25.5.

**Fig. 6:**
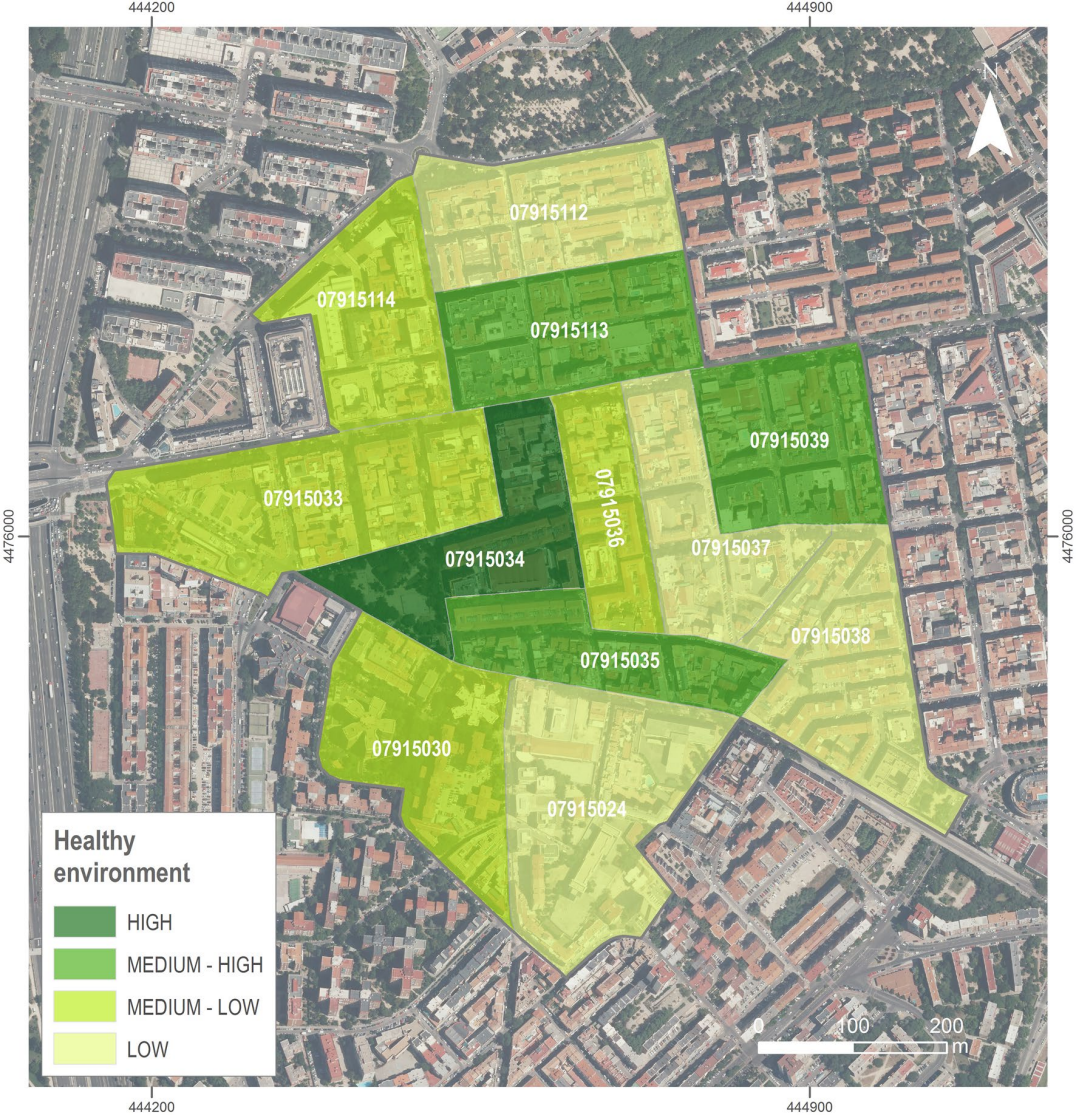
Study area characterization at census section level. Is the result of zonal analysis of each administrative area having in account all the pixels of the local analysis within each area

Another result is obtained from zonal analysis, where we mapped the 12 census sections of the area in Fig. 6 with single output value for each census section averaging all pixels that fall within each area. Characterization created by using natural Jenks grouped the census sections into four categories about themselves according to the average score: low (17.7–21.6), medium–low (21.7–30.8), medium–high (30.9–35.1) and high (35.2–43.8). Four out of the 12 census sections are classified as low, 4 medium–low, 3 medium–high and 1 as high.

 Table 1 shows basic sociodemographic characteristics of the twelve census sections and the entire study area. Around 4.2 % of the population live in areas characterized as “healthy” environments (defined as a “high” synthetic index), while 41.0 % of the residents live in an area with the lowest rating. Analysing the results by sex, 40.8 % of women have lower scores than men (41.3 %). 4.6 % of women and 3.7 % of men live in a section with high score. In the case of foreign-born residents, 49.3 % of them live in the unhealthiest areas and 3.0 % in the healthier. If results are studied by age, the majority of young people live in a census section with low score (51.5 %) as well as adult people (44.4 %) but in the case of elderly people, they live in a high healthy space (27.7 %). Only the 1.9, 3.5 and 7.0 % of young, adult and elderly people respectively live in a healthy section.

**Table 1:** Description of study area population by census section and group. Source: 2011 census data

Census section	7915024	7915030	7915033	7915034	7915035	7915036	7915037	7915038	7915039	7915112	7915113	7915114	Total
Population (%)	980 (6.5)	1110 (7.4)	1265 (8.4)	635 (4.2)	1205 (8.0)	540 (3.6)	133 (8.9)	2145 (14.3)	1980 (13.2)	1680 (11.2)	1480 (9.9)	625 (4.2)	14,980
Women (%)	555 (6.5)	600 (7.0)	780 (9.1)	395 (4.6)	670 (7.8)	320 (3.7)	750 (8.7)	1210 (14.1)	1135 (13.2)	980 (11.4)	865 (10.1)	315 (3.7)	8575 (57.2)
Foreign born (%)	130 (7.1)	150 (8.2)	70 (3.8)	65 (3.6)	60 (3.3)	65 (3.6)	125 (6.8)	395 (21.6)	440 (24.1)	405 (22.2)	180 (9.9)	55 (3.0)	2140 (14.3)
Years < 16 (%)	180 (13.7)	60 (4.6)	135 (10.3)	25 (1.9)	130 (9.9)	70 (5.3)	125 (9.5)	230 (17.6)	105 (8.0)	140 (10.7)	40 (3.1)	70 (5.3)	1310 (8.8)
Years > 65 (%)	180 (4.9)	310 (8.5)	435 (11.9)	255 (7.0)	300 (8.2)	160 (4.4)	250 (6.9)	350 (9.6)	505 (13.9)	230 (6.3)	395 (10.8)	275 (7.5)	3645 (24.3)
Area in km^2^ (%)	0.05 (11.9)	0.04 (9.5)	0.05 (11.9)	0.03 (7.1)	0.03 (7.1)	0.02 (4.7)	0.03 (7.1)	0.04 (9.5)	0.03 (7.1)	0.03 (7.1)	0.03 (7.1)	0.03 (7.1)	0.42
Pop. density inhab/km^2^	19,600	27,750	25,300	21,167	40,167	27,000	44,500	53,625	66,000	56,000	49,334	20,834	35,667
Healthy environment	Low	Medium–low	Medium–low	High	Medium–high	Medium-Low	Low	Low	Medium–high	Low	Medium–high	Medium–low	

## Discussion

This paper documents the development of an innovative method to assess the obesogenic environment by using a synthetic index that integrates continuous measures of both food and physical activity environments generated by KDE. The results show a heterogeneous distribution of obesogenic determinants in the study area. 36.5 % of the census sections have a low synthetic index value, followed by medium–low healthy (33.7 %), and medium–high (22.4 %). Only one census section falls under the category of high value in the synthetic index, representing only 4 % of the study area.

This healthy census section is delimited by the main streets of the neighborhood, where healthiest food stores were present. Main streets are also designed to be more walkable and have more intersections. Moreover, while we did not measure parks, the only park located in the study area, was also located within this census section. The census sections with the lowest synthetic index value were located in the east of the study area. Food store density is smaller with 3 corner stores, 1 bakery and 1 small supermarket, and we found narrow streets residential inter-block.

In order to understand the obesogenic environment it is necessary to consider the interrelations between the food and physical activity environments, as built environment metrics are correlated with each other [44]. The use of composite indices reduces collinearity and over-adjustment, confers ease of interpretation, and may reduce measurement errors [18]. Besides, integrating different indicators within an index can detect associations not previously found [28]. In our case, a systematic observation of a built environment, using validated audit tools, provided highly detailed spatial data. This also ensured variability on measures of both constructs and statistical power. The use of an extensive sampling strategy to maximize the variation between environmental factors reduced the sample sized needed to assess associations between built environments and obesity outcomes [45].

Previous studies have considered both physical activity and food environments to characterize environmental obesogenity, but have not obtained a composite score [25, 27, 45]. These urban environment measures have been used to evaluate their relation with diabetes incidence [46] or cardiometabolic risk factors [47]. The method described here considers previously studied variables, such as food store density, food store type, street intersection density, parks or street aesthetic, among others. The majority of these studies used GIS to integrate all information from diverse sources, mostly from secondary databases. On top of these, our study adds other variables as availability of healthy foods captured by NEMS-s or the aesthetic or safety domains measured by M-SPACES tool, which are very difficult to assess from secondary administrative databases.

KDE remains underutilized when compared to proximity analysis or to analysis over defined statistical areas [48–51], although the number of examples using KDE technique to study the obesogenic environment has increased in recent years [48–51]. KDE overcomes the limitations of binary definitions present in analysis based in fixed geographic boundaries (for example, number of stores per census section). Smooth transitions across (administratively defined) boundaries represent the reality of urban environments better [52]. The resulting KDE surface can then be used as an independent variable on statistical models [42].

Our study was conducted in the City of Madrid, Spain. In Spain, the smallest administrative level where data is publicly available is the census section, composed of ≈1500 people. Our study area is made up of 12 census sections, although the estimation of our synthetic index creates a smoothed surface over the entire study area, regardless of census section boundaries. This method is therefore replicable in other settings where the administrative spatial hierarchies are different, as long as data is collected at the appropriate level. This method is also replicable at larger units, like municipalities or countries, taking always into account the effort associated with data collection at any level.

The proposed method has several limitations. First, it requires primary data collection through systematic observation, which is a resource and time intensive process. Thanks to advances in Geographic Information Technologies, these costs can be drastically reduced, by using available secondary databases with spatial information and new geographic remote devices to collect geocoded primary data [29]. Second, this work has not considered the relative importance of the two domains with respect to each other, treating both environments with equal weight. The controversy regarding the quantification of the proportion of food or physical activity responsible for the obesity epidemic is still very much alive [53, 54]. We decided to adopt a local unweighted average, but any study using this method to estimate the associations between obesogenic environment and health outcomes should consider sensitivity analysis that alter these weighting decisions.

## Conclusion

The proposed synthetic index provides a feasible way to integrate different measures of physical barriers and promoters of healthy urban environments. This method opens new ways to capture inter-relations between physical activity and health food availability urban environment domains that did not emerge when they were studied in an isolated way. Thus, applying this index is a preliminary step to promote healthier urban environments and bridge the health inequalities present in large cities like Madrid.

The proposed index, and the cartography associated with it, may be useful tools to inform future research and urban health recommendations.

## Abbreviations

GIS: Geographic Information System
MNI: Median Neighborhood Index
NEMS-S: Nutrition Environment Measures Survey in Stores
HFAI: Healthy Food Availability Index
M-SPACES: Madrid Systematic Pedestrian and Cycling Environment Scan
KDE: kernel density estimation
